# Strictura Recti. Linear Rectotomy—Cure

**Published:** 1880-02

**Authors:** Herman Mynter


### STRICTURA RECTI. LINEAR RECTOTOMY—CURE.

REPORTED BY HERMAN MYNTER, M. D.

MRS. S., forty years of age, consulted me in December, 1877. In 1871 she experienced difficulty in defecation; two years after a swelling, accompanied with great pain, came around the anus, resulting in suppuration and the formation of several fistulas, which have since continually kept open, other fistulas occasionally forming. The impediment to defecation increased steadily, and for four years an evacuation of the rectum has taken place only by the use of cathartics; at times complete occlusion would occur with great meteorrhismus, pain and vomiting, lasting for several days. Gradual dilatation with bougies has been resorted to, but with only temporary relief; no symptoms of syphilis were apparent; healthy children were borne; the husband had venereal disease which yielded to potass. iodid.

On examination the anus was almost encircled with fistulas, the skin and subcutaneous textures indurated; three-fourths of an inch above the anus, almost a perfect diaphragm of the rectum was discovered, with a small opening anteriorly near the vagina, through which a bougie, 26 millimetres in circumference, could be introduced; the stricture was hard as cartilage. A sound introduced through the fistula was felt under the mucous membrane, until it disappeared behind the stricture. The general health from constant anxiety, the frequently recurring rectal occlusions, and the strict dietary observed to avoid evacuation of the bowels, had become very much impaired.

Through the application of electrolysis for a period of three weeks, a knob 36 millimetres in circumference was passed with considerable ease through the stricture, the negative pole, a metallic electrode, being introduced into the stricture while the positive pole with a moistened sponge was applied to the sacrum; a current from ten or fifteen cells was used; the applications were so painful that at the end of the third week the treatment was omitted, although it had so far dilated the stricture that defecation was accomplished with greater ease and facility; large doses of potass. iodid were used.

In January, 1879, the patient consulted me again; her previous symptoms had returned with all their severity, and she expressed a willingness to submit to any operation to gain relief; the stricture had contracted so that a bougie of 18 millimetres in circumference only could be introduced; new fistulas had formed; increased amaciation was observed.

January 11, 1879. Operation under chloroform; Drs. Gay and Lothrop assisting. The sphincter ani was divided by means of a galvano-cautery battery, the loop being introduced through one of the fistulas and brought out through the anus. The index finger and several uterine dilators failed to dilate the stricture. Every attempt to carry the wire above the stricture—through the fistula—was unsuccessful; the stricture was at length incised with herniotome, and all the contracted tissues freely cut through until two fingers could be easily passed; the mucous membrane above the stricture was ulcerated. A plug saturated with carbolic acid and sweet oil (1 to 10) was applied, and the rectum syringed out with carbolized water; anodynes prescribed to relieve pain. Convalescence was rapid, the parts healing rapidly and in three weeks the patient was discharged; considerable power had been regained on the sphincter ani and two fingers could be readily introduced. A large rectal bougie has since been used daily, the fistulas have healed, the general health improved, and evacuations of the rectum take place without pain and with comparative ease. No further contraction has since taken place.

